# The interaction between dietary and life goals: using goal systems theory to explore healthy diet and life goals

**DOI:** 10.1080/21642850.2014.927737

**Published:** 2014-07-15

**Authors:** Gabrielle M. Turner-McGrievy, Julie A. Wright, Jeffrey P. Migneault, Lisa Quintiliani, Robert H. Friedman

**Affiliations:** ^a^ Department of Health Promotion, Education, and Behavior, University of South Carolina, Columbia, SC29205, USA; ^b^ College of Nursing and Health Sciences, University of Massachusetts-Boston, Boston, MA02125, USA; ^c^ General Internal Medicine, Boston University School of Medicine, Boston, MA02215, USA; ^d^ Department of Medicine, Boston University School of Medicine, Boston, MA02118, USA

**Keywords:** goal setting, health behavior theory, diet

## Abstract

*Objective*: To examine the types of life and dietary goals individuals report and how these goal domains interact as framed by goal systems theory. *Methods*: This work is a cross-sectional survey study. Measures included the incidence of common life and dietary goals and how these goals interact with and facilitate each other. *Results*: The results of a quantitative survey (*n* = 46 participants), which was informed by two focus groups (*n* = 17 participants), showed that participants are trying to achieve several different life (e.g. achieving financial success) and dietary goals (e.g. eating more fruits and vegetables, drinking more water, and losing weight) and that these two types of goals interact to both facilitate and conflict with each other. Having a life goal of exercising was significantly associated with healthy eating goals when compared with other life goals (*p*'s < .05), suggesting these goals may be linked and help to facilitate one another. Being in the maintenance phase with the goal of healthy eating was associated with participants feeling like they were more successful in their other non-diet-related health goals (*p* < .05), suggesting maintenance of goals can facilitate success in achieving other goals. *Conclusions*: Life goals can have an impact on a person's ability to achieve and maintain dietary and other health goals. Health educators may help to facilitate long-term behavior change by examining a person's life goals as well as dietary goals.

Although many dietary interventions have shown some success, the objective of long-lasting dietary change has not been broadly or reliably achieved (Butryn, Webb, & Wadden, 2011; Jehn, Patt, Appel, & Miller, 2006; Teixeira et al., 2004). Finding successful ways of promoting long-term maintenance is important if dietary interventions are going to have a significant health benefit. Many behavioral interventions have employed the use of goal setting as part of a self-regulation strategy (Schnoll & Zimmerman, 2001), which has been shown to be one of the most effective ways to promote health behavior change (Nothwehr & Yang, 2007; Shilts, Horowitz, & Townsend, 2004). It is clear that dietary change over the long term requires incorporation of healthful eating goals into one's overall everyday lifestyle. While research on diet and other health-related behavior change has focused on factors that promote and impede initiation of behaviors (e.g. lack of available time, limited financial resources, and presence of supportive family/friends) (Lucan, Barg, Karasz, Palmer, & Long, 2012; Lucan, Barg, & Long, 2010), there has been less research on how life goals (e.g. being a good parent, maintaining employment) can be used to facilitate the achievement of dietary goals and how to identify and manage other life goals that may conflict with dietary goals. Furthermore, and central to this study, little is known about how people balance their dietary goals with their other life goals, which may be key to understanding how goal setting can be used to promote maintenance of dietary behavior change.

Goal systems theory (GST), proposed by Emmons and King (1988), Karoly (1991), Kruglanski et al. (2002), Shah, Friedman, and Kruglanski (2002) and Shah, Kruglanski, and Friedman (2002), is a potential model for understanding how goals relate to each other and provides a potential framework to describe how varying levels of motivation can be influenced by interrelated goals (Kruglanski et al., 2002). GST assumes that goals, as cognitive constructs, are mentally represented and organized and that this organization may help determine how goals are chosen and pursued over time. Individuals can have primary goals, which are in turn influenced by sub-goals and various means to achieve those sub-goals (Kruglanski et al., 2002). According to GST, goals may be combined to achieve one larger overarching goal. Data have shown that “integrating” goals (such as seeing how a life goal of saving money is complementary to a dietary goal of reducing consumption of soda) can lead to positive health outcomes (Affleck et al., 1998; Emmons, 1991; Emmons & King, 1988; Karoly, 1991; Sheldon & Kasser, 1995). There are four domains of effective goal management described in the GST literature (Emmons & King, 1988; Karoly, 1991; Shah, Friedman et al., 2002; Shah, Kruglanski et al., 2002): (1) finding ways to reduce conflict between competing goals (i.e. “goal conflict reduction”; Emmons & King, 1988); (2) applying strategies that use other goals to help attain diet goals (i.e. “cross goal facilitation”; Doest, Maes, Gebhardt, & Koelewijn, 2006); (3) finding ways to protect or shield the targeted goal from other competing goals (i.e. “goal shielding”; Shah, Friedman et al., 2002); and (4) addressing ways to maintain the resources that are necessary for the particular goal (i.e. facilitating goal maintenance; Jong, 2001).

The present study examines the constructs of goal facilitation, goal conflict, and goal maintenance. GST posits that life goals can be used to help facilitate diet goals (i.e. “cross goal facilitation”; Doest et al., 2006) but also conceptualizes goal conflict as the competition between goals for limited resources. With regard to goal maintenance, achieving a goal and maintaining that goal may help to facilitate self-efficacy and attainment of other goals (Schunk, 1990). In relation to maintenance of dietary behavior change, the central premise of our perspective is that maintenance of health behavior change may fail because of reasons outside of the domain of the specific health behavior, namely, competition from other life goals.

There is a dearth of information on the types of lifestyle goals (including dietary goals) individuals have. The aim of this study was to explore and describe the types of goals individuals have with the purpose of informing survey and intervention development for a larger trial. This cross-sectional survey study explores the inter-relationships between a person's lifestyle goals: dietary and other life goals (hereafter referred to as diet goals and life goals). While diet goals could potentially be a subset of overall life goals, the present study aimed to see how goals outside of diet-related goals could be used to facilitate the achievement and maintenance of diet goals or can impede dietary goal achievement. In turn, this information can be used to help improve diet-related interventions by including other non-diet-related goals. We hypothesized that life goals which are more closely tied to improving health (e.g. goal to exercise) would be more likely to be associated with diet goal facilitation (e.g. goal facilitation), whereas other life goals may be associated with greater diet goal conflict (e.g. goal conflict). In addition, we hypothesized that those actively maintaining a diet goal, versus seeking to achieve a diet goal, may be more successful in achieving life goals (e.g. goal maintenance).

## Methods

Adults were recruited to participate in a one-time survey. Recruitment took place in a restaurant-cafe on the first floor of an office building in the city of Boston. Customers were invited to complete an anonymous survey and were offered a $5 gift certificate from the restaurant. The only inclusion criteria was age (>18 years). The research protocol was approved by the Boston University Medical Center Institutional Review Board.

The survey was developed using literature on GST (Karoly, 1991; Shah, Kruglanski et al., 2002), published surveys with content validity (Karoly & Ruehlman, 1995; Karoly et al., 2005), and results from two focus groups recruited from advertisements placed in a local, free newspaper in inner-city Boston. The purpose of the groups was to assist in generating items for the survey. Focus groups were asked to discuss their goals and types of goals and explore how these types of goals interact. Both focus groups were conducted by two trained researchers (facilitator and a note taker), and a discussion guide was used to guide the facilitator. The sessions began with a group discussion of the participants' “life goals”, “needs”, “life priorities”, and “major life activities”. This discussion helped to generate relevant life and diet goals for the survey. Next, participants were given a list of 49 common life goals taken from existing literature from which they were asked to individually select goals (by circling them) they were currently trying to achieve by working on them at least weekly. They were asked to add any goals they had that were not on the list. Finally, participants indicated their five most important goals. A similar process was used for dietary goals (20 total, including “eat more fruits and vegetables”, “cut back on salty foods”, etc.). This process helped to narrow the list of potential life and diet goals for the survey. Next, the facilitator led a discussion with the whole group about what goals were selected, how often participants think about these goals, and their perceptions of how goals interrelate. Participants were then asked to write down their most important dietary goal and their top five life goals from the previous exercises, and rate how much these five life goals interfered or assisted with achieving their dietary goal (7-point scale with 1 = life goal made achieving success on the dietary goal much more difficult and 7 = life goal made it much easier). This handout was returned to the facilitator. Lastly, the facilitator led a group discussion on the interaction of life and dietary goals, guiding participants to discuss how their life goals both interfered and assisted with meeting their dietary goals. This last exercise helped to create survey questions around how diet and life goals may influence one another.

### Survey

Based on the results of the focus groups, a survey was developed to assess current life goals, dietary goals, and how these goals influence each other. The paper–pencil survey, which has not been used in prior studies, comprised 11 pages with 5 pages devoted to the present study's purpose and 6 pages allocated to a separate survey study about one's physical neighborhood environment in relation to walkability and food stores (those results are not reported here). The relevant portion of the survey assessed current dietary and life goals, and how all of these goals influenced each other. The most common dietary goals were identified by asking participants to identify the dietary goals they were currently working on from a checklist of 24 dietary goals. Participants were instructed to check only the goals they were actively trying to achieve (i.e. goals they were working on at least weekly).

Space was included for participants to write in their own goals if they were not included on the checklist. Participants were asked to identify the one dietary goal most important to them. The same checklist format was used to identify the most common life priorities, goals, or activities. Participants put a check mark next to any of the 20 goals on the checklist that they perceived as important and spent time on them at least weekly, and were asked to circle their two most important goals on the list. Success in meeting life goals was assessed on a 7-point scale from 1 (no success), 4 (moderate success), to 7 (total success). Participants were then asked to select the two life goals that were most important to them and then select one of those two goals to focus on in order to assess how this life goal interacts with their dietary goals. Goal conflict and facilitation was assessed by asking how their most important life goal affected the achievement of their most important dietary goal with the question, “Overall does this goal make being successful on your diet goals harder or easier” rated on a 7-point scale from 1 (much harder) and 7 (much easier). Goal maintenance was assessed by looking at the relationship between having an active diet goal and a maintenance diet goal. Goal shielding was not assessed.

### Statistical analysis

Descriptive statistics were used to explore the frequency goals that were endorsed and mean ratings on the interval scales. Independent *t*-tests and analysis of variance (ANOVA) were used to examine group differences with Tukey's honestly significant difference (HSD) test for *post hoc* analyses when appropriate.

#### Goal facilitation

ANOVA was used to examine whether life goals differ on their facilitation of healthy eating goals. Goal facilitation ratings (e.g. does this goal make it easier or hard to achieve diet goals) were compared across the top four life goals.

#### Goal maintenance

It was hypothesized that those who were successful at maintaining a dietary goal would have higher success on their life goals. Independent *t*-tests were used to examine differences in ratings of perceived success in life goal achievement between those who endorsed maintaining a dietary change as their most important diet goal and those who selected actively working toward a dietary goal as their most important.

All analyses were conducted using statistical package for the social sciences 21.0 for Windows software (Armonk, NY) with a *p*-value of .05 used to indicate statistically significant differences.

## Results

### Focus groups for survey development

The goal of the focus groups was to generate a list of common life and diet goals to be used in a quantitative survey. A total of 17 adults participated in the focus groups. The first focus group consisted of four men and five women who reported being employed (six African-American, two White, and one Hispanic). The second group consisted of eight women who reported having children (four African-American, three White, and one Hispanic). The participants discussed a variety of goals including becoming more financially secure, planning for retirement, being healthy, having fun, following your passions, getting ahead, and helping kids. After reviewing the list of 49 life goals to which participants could endorse as important, participants selected their top 5 most important goals.

When participants were asked about dietary goals, many responses were related to losing weight. Participants mentioned goals such as carbohydrate counting, reading food labels, and reducing sodium, sugar, or fat intake. When given the list of 20 dietary goals, participants chose goals such as eating more fruits and vegetables, drinking more water, using less oil, taking vitamins, and eating more whole grains as their most important dietary goals.

In terms of how life and dietary goals and available resources conflicted with and facilitated each other, the most common responses from the two focus groups were financial constraints to buying healthy foods and time constraints to purchase healthy foods and maintain a healthy lifestyle. Participants also mentioned availability and transportation conflicts as barriers to healthy eating. In addition, participants cited as goals of achieving financial success and goal of maintaining a house as goals which can interfere with healthy eating and exercise goals. Overall, participants provided fewer examples of goal facilitation compared to goal conflict. One example of goal facilitation was that meeting the goal of having reliable employment facilitated the goal of buying healthier foods. In addition, although not referring to a dietary goal, participants discussed how life goals can work together in order to make both goals stronger, such as achieving both goals of stress relief (a life goal) and getting healthy (health goal) through regular physical activity.

### Frequency of diet and life goals

Demographics of the survey participants (*n* = 46) are presented in Table 1. Participants had a mean age of 38.2 (range 21–73 years) and 76% were White with 83% having a college degree. Table 2 shows the percentage of participants who chose each dietary goal and the percent who endorsed that goal as the most important. The most common goal was to “Drink more water” (74%) followed by “Eat more fruits” (59%). Weight loss (24%) and maintaining a dietary behavior (13%) were the two most frequently chosen as a participant's most important dietary goal. Twenty-eight percent of participants did not report a dietary goal as most important.

**Table 1.** Demographics for survey participants (*N* = 46).

Characteristics	Mean
Age	38.2 (range 21–73)
*Marital status*	Frequency^a^
Single	33%
In a relationship	24%
Married	41%
*Children in the household*	
Children under 12	29%
Children 12–18	16%
*Education*	
High school	7%
Two-year college	9%
Four-year college	37%
Graduate degree	46%
*Employment*	
Full time	85%
Part-time	9%
Unemployed	5%
*Income*	
<$10,000	4%
$10,000–30,000	11%
$30,000–50,000	13%
>$50,000	50%
*Race/ethnicity*	
Asian	5%
Black or African-American	13%
Native American	3%
White	76%
Other	3%
*Hispanic*	
Yes	5%
No	95%

^a^Percent amounts do not always add up to 100% because some participants chose not to answer some of the demographic questions.

**Table 2.** Survey results: percentage of survey participants who selected each dietary goal and the frequency of which dietary goals were selected as being the most important (*n* = 46).

Dietary goal	Frequency chosen as a current dietary goal (%)	Frequency chosen as most important dietary goal (%)
Drink more water	74	6
Eat more fruits	59	2
Eat less sweets	54	7
Eat more vegetables	50	2
Lose weight	50	24
Maintain healthy changes that I have already made to my diet	48	13
Eat a balanced diet	35	7
Eat less fast food/take-out	33	4
Take daily vitamins	33	0
Eat smaller portions	33	0
Eat more whole grains	30	0
Eat less fried food	24	0
Drink less soda or other sugar-sweetened drinks	24	0
Cut down on salt	20	0
Get recommended daily amount of calcium	20	0
Drink less caffeine	11	0
Stop skipping as many meals	9	0
Stick to a diet which has been prescribed to me by my healthcare provider	9	0
Eat less meat	4	0
Stick to a special diet	4	0
Drink less (alcohol like beer, wine, hard liquor)	4	2
Gain weight	2	0
I do not have any of the dietary goals listed here	2	0
Stop drinking alcohol	0	0

Table 3 shows the percentage of participants who chose each life goal and the percentage who endorsed that goal as the most important. The most common life goal was to “Save money or invest for the future” (80%) followed by “Exercise regularly” (78%). The four most frequently chosen top goals were “Exercise regularly” (15%), “Be a good parent” (14%), “Save money or invest for the future” (13%), and “Spend quality time with friends and/or loved ones” (11%) (2% of participants did not report a most important life goal). Participants on average rated their success in meeting life goals as 4.62 ± 0.91 with the majority (*n* = 31) responding in the middle (3–5).

**Table 3.** Survey results: percentage of survey participants who selected each life goal and the frequency of which life goals were selected as being one of the two most important goals (*n* = 46).

Life goal	Frequency chosen as current life goal (%)	Frequency chosen as most important life goal (%)
Save money or invest for the future	80	13
Exercise regularly	78	15
Be well dressed and have a pleasing appearance/look good	74	3
Spend quality time with friends and/or loved ones	74	11
Improve or keep up the appearance of my home/apartment	70	1
Do well at work	61	4
Make time for the things I really enjoy doing (hobbies, music, reading, art, dancing, etc.)	54	6
Earn more money	52	6
Have more energy	46	6
Learn new things (take a course, attend a workshop, read a book)	44	4
Reduce the stress in my life	44	4
Be a good parent	37	14
Get more education/another degree	35	6
Help others in need	28	0
Work on improving my community	24	0
Take care of others in my extended family (parents, grandchildren, nieces, nephews, etc.)	24	2
Provide good food for my family	24	1
Be active in my church or place of worship	20	1
Play or compete in sports	20	0
Get a new or better job	17	2

### Goal conflict and facilitation

For the life goal participants identified as most important, participants were asked how this goal conflicts or facilitates with their dietary goal (i.e. “does having this goal make it harder or easier for you to achieve the dietary goals”). The mean response was 4.26 ± 1.43 (with the majority (*n* = 27) responding in the middle scoring 3–5) meaning, overall, participants reported that their most important life goal had no effect on achieving their dietary goals. The ANOVA comparing goal facilitation ratings (how well the life goals facilitated achieving their most important dietary goal) across the top four life goals (exercising regularly, good parent, save money, and spend time with friends) was significant (*F*(3, 44) = 8.13, *p* < .01). Follow-up Tukey's HSD tests showed that having a life goal of exercising regularly had significantly higher ratings than the other three life goals. Mean facilitation score for achieving dietary goals was significantly higher for exercise (5.5 ± 1.5) when compared with being a good parent (4.1 ± 1.1, *p* < .05), saving money (3.8 ± 1.0, *p* < .01), and spending time with friends (3.7 ± 1.2, *p* < .01).

### Facilitating goal maintenance

The effect of having an active diet goal (weight loss) or a maintenance diet goal (maintaining dietary changes already achieved, both of which were frequently prioritized as the top diet choice) on how successful the individual felt they were in achieving their life goal (goal maintenance facilitation) was also examined. Participants with a goal of maintaining healthy dietary changes (*N* = 6) reported being significantly more successful in meeting their other life goals (5.0 ± 0.0) than those who chose an active diet-related goal of weight loss (*N* = 10) (4.18 ± 0.87) (*p* < .05) although both reported at least moderate success.

## Discussion

The results of this study show that people report that they are actively working on important dietary goals. Finding ways to help people achieve and maintain these goals is important. This exploratory research project obtained data on important life (e.g. be financially successful and spend time with others) and dietary goals (e.g. losing weight, eating more fruits and vegetables, and eating a balanced diet) and how diet and life goals are perceived and interact. Practitioners, educators, and researchers designing diet-related interventions who focus on dietary change and goals (e.g. health psychologists, dietitians, and public health researchers) while limiting discussion or intervention components about a person's life goals may be missing an opportunity to facilitate long-term behavior change. Identifying life goals may help practitioners and researchers explore resistance to healthy eating (e.g. examine conflicts to healthy eating with one or more life goals) and then use strategies to integrate life and healthy eating goals together, such as examining integrated regulation from self-determination theory (Patrick & Williams, 2012) or applying strategies from motivational interviewing to resolve ambivalence or conflicts between goals (Britt, Hudson, & Blampied, 2004; Rollnick & Miller, 1995). Nutrition education and intervention, therefore, may be most effective when important goals – nutrition and otherwise – are discussed with patients and study participants.

This study provides a better understanding of the types of goals individuals have in both diet and life domains. While neither an exhaustive list of goals was studied nor was the study sample representative of the larger population, the respondents did endorse a cluster of goals more frequently. When asked to select the most important life goal, the most commonly prioritized goal was “exercising regularly” which was second only to “saving money”; while the most commonly prioritized dietary goals were “drinking water” and “eating fruit”. With the caveat that this population is mainly from a higher socioeconomic status (SES), they were only reporting moderate success on these goals. Population rates of meeting exercise and diet recommendations are low but typically higher among higher SES categories (Wang & Chen, 2011).

The dietary goal to lose weight was the most frequently rated as a top priority by study participants. This is not surprising considering that a quarter of men and one-third of women in the USA report that they are currently attempting to lose weight (Kruger, Galuska, Serdula, & Jones, 2004). The next most frequently chosen dietary goal was to maintain healthy choices that the participant already made. This study found exploratory evidence that those with a maintenance goal reported more confidence in their ability to achieve their other life goals than those with a goal of weight loss. Research with a larger sample size is needed for further exploration of this but there are several potential reasons for this relationship that could be hypothesized. For example, from a social cognitive theory perspective, this could be due to the fact that accomplishing a personal goal can often boost self-efficacy (Gist & Mitchell, 1992) and foster success in other areas. Future research using a GST perspective should explore if maintaining a dietary change takes fewer resources to achieve than achieving a new goal, therefore freeing up resources for a person to pursue another life goal.

One of the most interesting findings was that those who endorsed exercise as the most important had higher facilitation ratings for diet goals. This may suggest that exercising and healthy eating are linked as priorities or that achieving one makes it easier to achieve the other, both of which would reflect cross goal facilitation (Doest et al., 2006). Intervention research is examining the question of how to best present diet and exercise behaviors, either simultaneously or sequentially, to best capitalize on possible synergistic effects (King et al., 2013). While participants in the survey may not have seen ways that their life goals could help them meet their dietary goals, those with a health-related life goal (exercise) did see that goal as facilitating the dietary goal.

Goal facilitation can make meeting one's goals more efficient. For example, one study found that when work goals helped to facilitate personal goals, participants reported greater levels of well-being and job satisfaction (Doest et al., 2006). Often a goal of healthy eating is also associated with other health-related goals or a means to achieving another goal. For example, researchers found that healthy eating choices were made more often by people who had other health-related goals such as preventing disease, staying healthy, and improving the quality of life (de Almeida, Graca, Afonso, Kearney, & Gibney, 2001). While tying health-related goals to one another (e.g. healthy eating and preventing disease) is a natural fit, the benefit of identifying all life goals is to find ways that a goal of healthy eating may help facilitate the life goals that would seem otherwise disconnected. For example, having a goal of healthy eating may be seen as both a facilitator and a barrier for the life goal of saving money. Helping someone find ways to buy healthy foods (and buy fewer unhealthy foods) at a low cost can lower the barriers to healthy eating, increase the likelihood of meeting a savings goal, and improve dietary intake.

To the authors' knowledge, this is one of the first studies to examine both nutrition and life goals and how they interact with each other. One of the strengths of this study is the application of GST to population-based, health promotion research, which is novel. The limitations include the method of recruitment and the size of the sample. The survey sample was also not representative of the larger population as it was mostly well-educated, White, and had a higher SES than the national average. In addition, gender was not collected (an oversight which should be included on future administrations of the survey) therefore goal choice could not be examined by gender. This was a cross-sectional study so the effect of season or holidays could not be assessed. These different times of year may have varying impact on diet and life goals. For example, the goal to lose weight may become more prevalent during the New Year (Kassirer & Angell, 1998). In addition, goal shielding, a construct of GST, was not included. Future research should continue to examine all of the constructs of GST; however, valid and reliable measures of all GST constructs are necessary to investigate further how GST can inform and guide healthy eating interventions. The present study is limited in that it examined three of the four constructs and it was exploratory in nature. This study represents a way to conceptualize and elicit difficulties in attaining and maintaining healthy dietary habits that is better grounded in participants' daily life context than just focusing on barriers. Only focusing on barriers to nutrition-related goals may result in missed opportunities in identifying life goals as a facilitator in the achievement of dietary goals and does not acknowledge the positive nature of these other life goals and the difficult choices individuals face in allocating limited personal resources across life goals.
